# The Effect of Body Mass Index on Patients' Length of Stay Post-appendectomy

**DOI:** 10.7759/cureus.46430

**Published:** 2023-10-03

**Authors:** Muhanna A Alhusayni, Teif M Alghamdi, Wedad A Almutairi, Abdulaziz S Alhamyani, Fahad G Alosaimi, Mohammad Eid M Mahfouz

**Affiliations:** 1 College of Medicine, Taif University, Taif, SAU; 2 General Surgery, Taif University, Taif, SAU

**Keywords:** length of stay, appendectomy, obesity, body mass index, appendicitis

## Abstract

Background and aim: Appendicitis is defined as the appendix's inflammation, which requires an appendectomy for treatment. Obesity is one of the risk factors for post-surgical complications in appendicitis. This study aimed to explore obesity’s influence on hospital length of stay among patients with appendicitis in Taif, Saudi Arabia.

Methods: The study subjects consisted of both children and adults with appendicitis who were admitted to the hospital during 2021 and 2022. The patients were divided into three groups according to body mass index (BMI) following the WHO criteria: normal weight (BMI = 18.5-24.9 kg/m^2^), overweight (BMI = 25-29.9 kg/m^2^), and obesity (BMI ≥10 kg/m^2^). Data collection was conducted retrospectively by reviewing the medical records of patients diagnosed with appendicitis. The data collection included demographic characteristics of the patients, clinical presentation data, examination data, findings of the diagnostic approaches, management data, and complications after surgery (mainly intra-abdominal abscess formation). Prior to conducting the study, ethical approval was obtained from the Institutional Review Board of the Saudi Ministry of Health.

Results: The study included 238 patients who were diagnosed with appendicitis with an age range from 4 to 74 years and a mean (SD) age of 20.24 years (12.69). Based on BMI categories, 174 patients (73.1%) were classified as non-obese (BMI < 25), 53 patients (22.3%) were overweight (BMI 25-29.9), and 11 patients (4.6%) were obese (BMI ≥ 30). Most patients were presented with fever (51.7%), anorexia (72.7%), and pain in the right lower quadrant (70.2%). According to blood pressure, the mean (SD) of systolic and diastolic blood pressure was 117.8 (14.14) and 71.03 (9.89), respectively. All cases underwent abdominal ultrasound; non-visualization of the appendix was the most common finding observed (80.3%). Appendicitis was managed among most patients with appendectomy (89.5%), and open appendectomy was the most frequent surgery performed (73.2%). The mean of hospital length of stay was 1.64 (0.73) days. There was no significant correlation between patients’ BMI and hospital length of stay (p = 0.429). The mean hospital length of stay of females (1.76) was higher than that of males (1.53) (p = 0.003). Moreover, a statistically significant mean difference was observed in hospital length of stay between patients managed conservatively (1.96) and those who underwent operative management (1.60 days) (p < 0.001).

Conclusion: This study provides insights into the effect of obesity on patients with appendicitis who underwent an appendectomy in Taif, Saudi Arabia. The study found that obesity was not a risk factor for a prolonged hospital length of stay after appendectomy. Further studies with larger sample sizes are needed to explore other factors that may influence the outcomes of appendectomy in patients with appendicitis, such as the impact of obesity on long-term complications and recurrence rates.

## Introduction

Appendicitis is characterized by the inflammation of the vermiform appendix. It is recognized as a common and emergency surgical disorder requiring an appendectomy [1].

Appendectomy is one of the most frequent surgical procedures around the world, whether performed for acute appendicitis management or as an addition to other larger abdominal surgeries. Evidence in the United States showed that an appendectomy has been performed annually among 200,000 patients [2].

Appendectomy is classified into two types: open and laparoscopic appendectomy [3]. Open appendectomy may cause various complications, including adhesions, bowel obstruction, wound infection, intra-abdominal abscess, and pulmonary complications associated with general anesthesia. The mortality and morbidity rates associated with an open appendectomy were reported to be 0.3% and 11%, respectively. On the other hand, laparoscopic appendectomy has been shown to offer advantages such as quicker post-operative recovery, shorter hospital stays, and improved pain management, as highlighted in multiple studies [1].

The risks associated with appendectomy surgery in obese patients are generally low, and most patients recover without complications. However, obese patients may be at a higher risk of developing wound infections, respiratory complications, longer operating time, longer recovery time, and increased risk of blood clots. The recovery time may vary depending on the type of surgery performed, with laparoscopic surgery having a shorter recovery time of one to three weeks, while open surgery may take two to four weeks [4].

Although appendectomies are an emergency procedure that results in more challenging wounds and more significant post-operative discomfort in obese people, medical surgeons have to perform them more frequently. Researchers found that laparoscopic appendectomy can be operated safely in obese patients, with a shorter post-operative recovery [3].

Several studies highlighted the association between the patient's stay in the hospital after the appendectomy operation and the body mass index. A higher body mass index (BMI) increased the risk of the patient's stay in the hospital in a cohort of more than 800,000 hospitalized patients [3].

Nevertheless, a study conducted among 272 patients showed no significant change in the length of stay in the hospital in patients who were operated on for appendectomy besides the appendix perforation status and mortality rate among obese and non-obese patients [5].

Another study was conducted to compare the length of stay in the hospital between obese and non-obese patients. The frequency of any complication increased with the BMI category in the appendectomy cohort research. BMI percentage did not predict any specific complication or measurement of hospitalization in the intestinal surgery population-patients who were obese and had an appendectomy recovered longer in the hospital. Moreover, the duration of admission in hospitals was not significantly different for obese individuals who underwent intestinal surgeries [6]. However, there are few studies that evaluate its effects among patients in Saudi Arabia. The objective of this study is to examine obesity’s influence on hospital length of stay among patients with appendicitis in Taif, Saudi Arabia.

## Materials and methods

Study design

A retrospective study was conducted to explore the correlation between obesity and hospital length of stay among patients with appendicitis over two years (from 2021 to 2022). The study was conducted in King Abdulaziz Hospital and King Faisal Hospital, Taif City, Saudi Arabia.

Ethical approval

Prior to conducting the study, ethical approval was obtained from the Institutional Review Board of the Saudi Ministry of Health.

Study subjects

The study subjects consisted of children and adults diagnosed with appendicitis and admitted to the hospital between 2021 and 2022. Patients with incomplete or unreliable medical records were excluded.

Data collection

Data collection was conducted retrospectively by reviewing the medical records of patients diagnosed with appendicitis.

The data collection sheet included demographic characteristics of the patients, including age, weight, height, smoking status, past surgery history, and its type, and if there are other medical conditions, clinical presentation data, including fever, anorexia, constipation, diarrhea, original pain site, and the number of hours from the onset of pain, and examination data includes tenderness, location, rebound tenderness, Psoas signs, obturator signs, McBurney's signs, Rovsing's, guarding, systolic and diastolic blood pressure, and heart rate. Furthermore, other data were collected illustrating the findings of the diagnostic approaches, including X-ray, abdominal US, computerized tomography, magnetic resonance imaging (MRI), and complete blood count test (CBC), as well as management data, including type of management, type of appendectomy, duration of surgery, hospital length of stay, and complications after surgery (mainly intra-abdominal abscess formation).

Statistical methods

Data were extracted into an Excel sheet (Microsoft Corporation, New York) and then revised. The statistical analysis was conducted using the computer program IBM SPSS (version 26.0, Armonk, NY). Categorical variables were described in numbers and percentages. A normality test was performed for continuous variables. Patients were divided into three groups according to their BMI (normal <25, overweight 25-29.9, and obese ≥30 kg/m^2^), corresponding to the obesity value defined by the WHO [7]. The Mann-Whitney and Kruskal-Wallis tests were used to compare the hospital length of stay with the patients’ characteristics. A Spearman correlation test was used to identify the correlation between patients’ BMI and the length of stay. P-values less than 0.05 were considered statistically significant.

## Results

Table 1 presents the baseline characteristics of patients with appendicitis. The study included 238 patients who were diagnosed with appendicitis over two years (from 2021 to 2022). The age of the patients ranged from 4 to 74 years, with a mean (SD) of 20.24 years (12.69). The BMI of the patients ranged from 11 to 47, with a mean (SD) of 22.59 kg/m^2^ (5). Based on BMI categories, 174 patients (73.1%) were classified as non-obese (BMI < 25), 53 patients (22.3%) were overweight (BMI 25-29.9), and 11 patients (4.6%) were obese (BMI ≥ 30).

Most of the patients were male (n = 128, 53.8%), single (n = 211, 88.7%), and reported being non-smokers (n = 234, 98.3%). In terms of past surgical history, 17 patients (7.1%) reported having undergone previous surgeries, while the majority (92.9%) had no history of past surgeries. Among those with past surgical history, 16 patients (94.1%) had undergone open surgery before, while only one patient (0.4%) had a history of laparoscopy. A total of 20 patients (10.1%) reported having other medical conditions, while the majority (89.9%) had no additional medical conditions.

**Table 1:** Baseline characteristics of patients with appendicitis. BMI: body mass index, IQR: interquartile range, SD: standard deviation.

Age (years)	Median (IQR)	17 (17)	
	Mean (SD)	20.24 (12.69)	
	Min-max	4-74	
Height (cm)	Median (IQR)	158 (39)	
	Mean (SD)	149.8 (23.73)	
	Min-max	88-190	
Weight (Kg)	Median (IQR)	53.5 (35)	
	Mean (SD)	52.92 (22.19)	
	Min-max	15-150	
BMI	Median (IQR)	22.62 (6)	
	Mean (SD)	22.59 (5)	
	Min-max	11-47	
Parameters	Category	Total count (n = 238)	Percentage
Gender	Male	128	53.8
	Female	110	46.2
BMI	Non-obese (<25)	174	73.1
	Overweight (25-29.9)	53	22.3
	Obese (≥30)	11	4.6
Marital status	Single	211	88.7
	Married	27	11.3
Smoking	Non-smoker	234	98.3
	Smoker	4	1.7
Past surgical history	Yes	17	7.1
	No	221	92.9
Past surgery type	Laparoscopy	1	0.4
	Open	16	94.1
Other medical conditions	Yes	20	10.1
	No	214	89.9

Table 2 presents the baseline characteristics of patients with appendicitis categorized according to their BMI.

In terms of age, a small proportion of patients who were less than 18 years old and those with an age greater than 18 years old were classified as obese (2.5% and 6.8%, respectively). Regarding gender, among male patients, only five patients (3.9%) were classified as obese. Among female patients, approximately 5.5% were classified as obese. Among patients with a history of past surgeries, 11 (64.7%) had a normal BMI, 4 (23.5%) were overweight, and 2 (11.8%) were classified as obese.

Furthermore, regarding the presence of other medical conditions, among patients with other medical conditions, 18 (75%) had a normal BMI, 4 (16.7%) were overweight, and 2 (8.3%) were classified as obese.

**Table 2:** Baseline characteristics of patients with appendicitis regarding their BMI. BMI: body mass index.

Variables		BMI			Total
		Normal (BMI <25)	Overweight (BMI = 25-29.9)	Obese (BMI ≥30)	
Age (years)	<18	103 (85.8)	14 (11.7)	3 (2.5)	120 (50.4)
	≥18	71 (60.2)	39 (33.1)	8 (6.8)	118 (49.6)
Gender	Male	89 (69.5)	34 (26.6)	5 (3.9)	128 (53.8)
	Female	85 (77.3)	19 (17.3)	6 (5.5)	110 (46.2)
Marital status	Single	160 (75.8)	44 (20.9)	7 (3.3)	211(88.7)
	Married	14 (51.9)	9 (33.3)	4 (14.8)	27 (11.3)
Smoking	Non-smoker	173 (73.6)	51 (21.7)	11 (4.7)	235 (98.7)
	Smoker	1 (33.3)	2 (66.7)	0 (0)	3 (1.3)
Past surgical history	Yes	11 (64.7)	4 (23.5)	2 (11.8)	17 (7.1)
	No	163 (73.8)	49 (22.2)	9 (4.1)	221 (92.9)
Other medical conditions	Yes	18 (75)	4 (16.7)	2 (8.3)	24 (10.1)
	No	156 (72.9)	49 (22.9)	9 (4.2)	214 (89.9)

The clinical presentation, patients' examination findings, and diagnostic methods were described based on the patients’ BMI categories.

Regarding the clinical presentation, fever was observed in 73.2% of patients with a normal BMI, 23.6% of overweight patients, and 3.3% of obese patients. Anorexia was reported in 75.1% of patients with a normal BMI, 20.8% of overweight patients, and 4% of obese patients. Regarding gastrointestinal symptoms, constipation was reported by 79.8% of patients with a normal BMI, 18.2% of overweight patients, and 2% of obese patients. Diarrhea was present in 70.7% of patients with a normal BMI, 22.7% of overweight patients, and 6.7% of obese patients.

The mean (SD) onset of pain was 40.16 (41) hours. It was highest among obese patients, followed by overweight patients and those with a normal BMI (34.27 (18.25), 27.13 (14.06), and 17.26 (10.29) hours, respectively).

In terms of patients’ examination findings, tenderness in the right lower quadrant (RLQ) was observed in 72.6% of patients with a normal BMI, 22.4% of overweight patients, and 5% of obese patients. Rebound tenderness was found in 69.7% of patients with a normal BMI, 24.5% of overweight patients, and 5.8% of obese patients.

According to blood pressure, the mean (SD) systolic blood pressure was 117.8 (14.14) mmHg, and the diastolic blood pressure was 71.03 (9.89). Both systolic and diastolic blood pressure were highest among obese patients, with a mean (SD) of 133.45 (19) and 77 (10.33) mmHg, respectively.

In terms of diagnostic methods applied, non-visualization of the appendix was the most common observation in the abdominal US findings. It was seen more frequently among patients with normal weight (74.3%). Additionally, the mean (SD) of total leukocyte count was 10.69 (4.71), and it showed the highest mean (SD) among obese patients (11.06 (5.32)). Further details are described in Table 3.

**Table 3:** Clinical presentation, patients’ examination, and diagnostic methods among patients regarding their BMI. CBC: complete blood count, CT: computerized tomography, DBP: diastolic blood pressure, MRI: magnetic resonance imaging, RLQ: right lower quadrant, SBP: systolic blood pressure.

Variables		BMI						Total
		Normal (BMI <25)		Overweight (BMI = 25-29.9)		Obese (BMI ≥30)		
Clinical presentation								
Fever	Yes	90 (73.2)		29 (23.6)		4 (3.3)		123 (51.7)
	No	84 (73)		24 (20.9)		7 (6.1)		115 (48.3)
Anorexia	Yes	130 (75.1)		36 (20.8)		7 (4)		173 (72.7)
	No	44 (67.7)		17 (26.2)		4 (6.2)		65 (27.3)
Constipation	Yes	79 (79.8)		18 (18.2)		2 (2)		99 (41.6)
	No	95 (68.3)		35 (25.2)		9 (6.5)		139 (58.4)
Diarrhea	Yes	53 (70.7)		17 (22.7)		5 (6.7)		75 (31.5)
	No	121 (74.2)		36 (22.1)		6 (3.7)		163 (68.5)
Original pain site	RLQ	121 (72.5)		40 (24)		6 (3.6)		167 (70.2)
	Periumbilical	35 (70)		11 (22)		4 (8)		50 (21.0)
	Epigastric pain	10 (100)		0 (0)		0 (0)		10 (4.2)
	Other	8 (72.7)		2 (18.2)		1 (9.1)		11 (4.6)
Pain "shifts" to the right lower quadrant	Yes	48 (72.7)		13 (19.7)		5 (7.6)		66 (27.7)
	No	126 (73.3)		40 (23.3)		6(3.5)		172 (72.3)
Onset of pain (hours)	Median (IQR)	13 (14)		25 (25)		28 (34)		24 (24)
	Mean (SD)	17.26 (10.29)		27.13 (14.06)		34.27 (18.25)		40.16 (41)
Patients’ examination								
Tenderness location	RLQ	159 (72.6)		49 (22.4)		11 (5)		219 (92.0)
	Diffuse	13 (81.3)		3 (18.8)		0 (0)		16 (6.7)
	Other	2 (66.7)		1 (33.3)		0 (0)		3 (1.3)
Rebound tenderness	Positive	108 (69.7)		38 (24.5)		9 (5.8)		155 (65.1)
	Negative	66 (79.5)		15 (18.1)		2 (2.4)		83 (34.9)
Psoas signs	Positive	6 (60)		2 (20)		2 (20)		10 (4.2)
	Negative	168 (73.3)		51 (22.4)		9 (3.9)		228 (95.8)
Obturator signs	Positive	2 (66.7)		0 (0)		1 (33.3)		3 (1.3)
	Negative	172 (73.2)		53 (22.6)		10 (4.3)		235 (98.7)
McBurney's signs	Positive	1 (100)		0 (0)		0 (0)		1 (0.4)
	Negative	173 (73)		53 (22.4)		11 (4.6)		237 (99.6)
Rovsing's signs	Positive	12 (63.2)		6 (31.6)		1 (5.3)		19 (8.0)
	Negative	162 (74)		47 (21.5)		10 (4.6)		219 (92.0)
Guarding	Positive	3 (100)		0 (0)		0 (0)		3 (1.3)
	Negative	171 (72.8)		53 (22.6)		11 (4.7)		235 (98.7)
SBP (mmHg)	Median (IQR)	112 (13)		123 (16)		129 (20)		117 (15)
	Mean (SD)	114.9 (12.39)		124.06 (14.51)		133.45 (19)		117.8 (14.14)
DBP (mmHg)	Median (IQR)	70 (12)		72 (9)		75 (10)		70 (11)
	Mean (SD)	69.79 (9.53)		73.83 (10.12)		77 (10.33)		71.03 (9.89)
Heart rate (bpm)	Median (IQR)	88 (20)		88 (16)		86 (28)		88 (20)
	Mean (SD)	91.54 (15.12)		88.92 (11.15)		85.27 (15.98)		90.67 (14.41)
Diagnostic methods								
X-ray	Normal		7 (63.6)		3 (27.3)		1 (9.1)	11 (4.6)
	Dilatation of small bowel loops		1 (33.3)		2 (66.7)		0 (0)	3 (1.3)
	Not performed		166 (74.1)		48 (21.4)		10 (4.5)	224 (94.1)
Abdominal US findings	The diameter of the appendix >6 mm		5 (100)		0 (0)		0 (0)	5 (2.1)
	Free fluid surrounding the appendix		1 (100)		1 (100)		0 (0)	2 (0.8)
	Hyperechoic mucosa/submucosa		9 (100)		0 (0)		0 (0)	9 (3.8)
	Increased echogenicity of local mesenteric fat		0 (0)		1 (50)		1 (50)	2 (0.8)
	Non-compressibility of the appendix		14 (60.9)		8 (34.8)		1 (4.3)	23 (9.7)
	Non-visualization of the appendix		142 (74.3)		40 (20.9)		9 (4.7)	191 (80.3)
	Perforation compressible appendix		0 (0)		1 (100)		0 (0)	1 (0.4)
	Signs of secondary small bowel obstruction		1 (33.3)		2 (66.7)		0 (0)	3 (1.3)
CT findings	Normal		2 (50)		2 (50)		0 (0)	4 (1.7)
	Abnormal		2 (28.6)		2 (28.6)		3 (42.8)	7 (2.9)
	Not performed		170 (74.9)		49 (21.6)		8 (3.5)	227 (95.4)
CBC	Normal		114 (75)		31 (20.4)		7 (4.6)	152 (63.9)
	Abnormal		60 (69.8)		22 (25.6)		4 (4.7)	86 (36.1)
Total leukocyte count	Median (IQR)		9 (6)		9 (7)		9 (9)	9 (7)
	Mean (SD)		10.58 (4.76)		10.96 (4.49)		11.06 (5.32)	10.69 (4.71)
Pregnancy test	Positive		0 (0)		1 (100)		0 (0)	1 (99.1)
	Negative		85 (78)		18 (16.5)		6 (5.5)	109 (0.9)

Table 4 presents the management strategies employed and complications experienced by patients categorized based on their BMI.

Most patients underwent operative management in the form of appendectomy. Among them, 3.3% were obese patients. Regarding the appendectomy type, the open approach was more commonly performed across all BMI categories. In comparison, obese patients underwent laparoscopy more than open appendectomy (8.8% and 1.3%). Regarding the duration of surgery, the mean (SD) duration was 58.84 minutes (33.32). Moreover, it was higher among patients with an overweight BMI (70.28 (35.69)). The majority of patients did not experience complications after surgery. Particularly, no cases of abscess formation were reported among obese patients.

Furthermore, the mean (SD) length of stay in the hospital was 1.64 days (0.73), which was highest among obese patients (2 days (0.77)).

**Table 4:** Management and complications among patients regarding their BMI. BMI: body mass index.

Factors		BMI			Total
		Normal (BMI <25)	Overweight (BMI = 25-29.9)	Obese (BMI ≥30)	
Type of management	Operative (appendectomy)	155 (72.8)	51 (23.9)	7 (3.3)	213 (89.5)
	Conservative with antibiotics	19 (76)	2 (8)	4 (16)	25 (10.5)
Appendectomy type	Open	121 (77.6)	33 (21.2)	2 (1.3)	156 (73.2)
	Laparoscopy	34 (59.6)	18 (31.6)	5 (8.8)	57 (26.8)
Duration of surgery (minutes)	Median (IQR)	55 (30)	65 (45)	50 (95)	55 (40)
	Mean (SD)	55.55 (29.91)	70.28 (35.69)	55.91 (57.52)	58.84 (33.32)
Complications after surgery (intra-abdominal abscess formation)	Yes	2 (66.7)	1 (33.3)	0 (0)	3 (1.3)
	No	172 (73.2)	52 (22.1)	11 (4.7)	235 (98.7)
Hospital length of stay (days)	Median (IQR)	2 (1)	1 (1)	2 (0)	2 (1)
	Mean (SD)	1.66 (0.76)	1.51 (0.57)	2 (0.77)	1.64 (0.73)

There was a statistically significant association between hospital length and gender. The mean hospital length of stay of females (1.76) was higher than that of males (1.53) (p = 0.003). Moreover, a statistically significant mean difference was observed in hospital length of stay between patients managed conservatively (1.96) and those who underwent operative management (1.60 days) (p < 0.001).

Other variables, including age, BMI, smoking status, past surgical history, other medical conditions, and appendectomy type, had no significant impact on the hospital length of stay. Further details are described in Table 5.

**Table 5:** Hospital length of stay regarding patients’ characteristics. *Mann-Whitney test, **Kruskal-Wallis test.

Factors		Hospital length of stay		P-value
		Median (IQR)	Mean (SD)	
Age	Children	2 (1)	1.62 (0.7)	0.704*
	Adults	2 (1)	1.66 (0.76)	
Gender	Male	1 (1)	1.53 (0.73)	0.003*
	Female	2 (1)	1.76 (0.71)	
BMI	Normal	2 (1)	1.66 (0.76)	0.114**
	Overweight	1 (1)	1.51 (0.57)	
	Obese	2 (0)	2 (0.77)	
Smoking	Smoker	2 (1)	1.33 (0.57)	0.448*
	Non-smoker	2 (1)	1.64 (0.73)	
Past surgical history	Yes	2 (1)	1.82 (0.63)	0.138*
	No	2 (1)	1.62 (0.73)	
Other medical conditions	Yes	1 (1)	1.38 (0.49)	0.062*
	No	2 (1)	1.67 (0.74)	
Type of management	Conservative	2 (1)	1.96 (0.2)	<0.001*
	Operative	1 (1)	1.60 (0.76)	
Type of appendectomy	Laparoscopy	2 (1)	1.67 (0.80)	0.334*
	Open	1 (1)	1.58 (0.74)	

Regarding the Spearman test (Table 6), there was no significant correlation between BMI and hospital length of stay (p = 0.429).

**Table 6:** Association between patients’ BMI and their hospital length of stay. **Spearman test.

		Length of stay	P-value
Body mass index (BMI)	Correlation coefficient	-0.052	0.429**

## Discussion

Appendectomy is a common surgical procedure that is performed around the world. It is performed to manage acute appendicitis or as an adjunct to larger abdominal surgeries. Notably, obesity can play a crucial role in influencing patient morbidity in cases where an appendectomy is performed to manage acute appendicitis [8].

This study aimed to examine the effect of obesity among patients with appendicitis who underwent an appendectomy in Taif, Saudi Arabia.

Appendicitis typically manifests with an initial generalized or periumbilical abdominal pain that later becomes localized to the right lower quadrant. This pain may or may not be accompanied by additional symptoms, including anorexia, nausea, vomiting, fever (40% of patients), and diarrhea [9]. The clinical diagnosis of appendicitis is challenging and involves findings of clinical diagnosis, laboratory tests, and radiological examination [10]. In the current study, anorexia and fever were the most frequent symptoms observed among the patients. In comparison, most cases suffered from pain in the right lower quadrant. Moreover, abdominal US was the most frequent radiological examination performed, while CBC was the only laboratory test used to diagnose appendicitis.

It is widely recognized that overweight and obesity are well-established risk factors in all patients associated with several comorbidities. In addition, surgical procedures in obese patients tend to be more complicated due to the increased technical and anesthetic challenges. Furthermore, these patients' excess visceral adipose tissue accumulation leads to a pro-inflammatory state, resulting in metabolic changes that can affect the post-operative immune response and contribute to certain complications following surgery. Several studies have indicated a higher incidence of post-surgical complications among obese patients, such as surgical wound infection, as well as an increased mean hospital stay [11].

Particularly, wound infection was indicated to be the most common complication of appendectomy [1]. In our study, a small proportion of the cases reported complications (abscess formation) after surgery (1.3%). Unexpectedly, obese patients did not have complications after surgery. This may be attributed to the surgical team's expertise and their experience in managing obese patients. In addition, appropriate post-operative care leads to better surgical outcomes and fewer complications. On the contrary, Deugarte et al. [12] found most complications, particularly wound infections, among obese patients (9.1%). In addition, other studies found that complications after surgery, including intra-abdominal abscess, wound infection, wound disruption, and dehiscence, were statistically higher in overweight and obese patients compared to patients with a normal BMI [4,11,13].

In the current study, the mean hospital stay was 1.64 days, which was the highest among obese patients (two days). However, there was no significant mean difference regarding hospital stay among normal, overweight, and obese patients. Additionally, we explored the correlation between the BMI of the patients and hospital stay, and we found that there was no significant correlation between them (p = 0.429). Similarly, a previous study found that there was no significant mean difference between normal, overweight, and obese patients regarding post-operative length of stay (0.97 vs. 1.53 vs. 1.14 vs. days, P = 0.214) [14].

Contrary to our results, Garey et al. [13] and Knott et al. [15] indicated the presence of associations between obesity and a longer length of stay after appendectomy. Those studies also reported longer operative times and worse outcomes in obese appendectomy patients. Another study found that the mean length of stay was quietly higher (2.61 days) than our results, and obese patients had a significantly higher length of stay than others (p = 0.002) [16].

In this study, the gender affected significantly the hospital stay, where females tended to spend more days than males (p = 0.003). On the difference, Alharbi et al. [1] and Bhanderi et al. [17] found that males spent more days than females (p = 0.011 and 0.029, respectively).

According to a previous study, conservative antibiotic treatment has been found to be a potentially effective approach for patients with acute appendicitis, suggesting that surgery may not always be necessary in these cases. Furthermore, patients managed conservatively with antibiotics alone experienced lower pain levels and required less analgesia. Despite these benefits, this conservative approach has a higher recurrence rate. In addition, the mean hospital stay was higher among patients treated with antibiotics than others who underwent surgery (2.3 vs. 1.2 days) [18]. Our findings supported these results and found that patients who managed conservatively reported a longer hospital stay than those who underwent operative management (1.96 vs. 1.6 days, respectively) (p < 0.001).

Limitations

This study had some limitations, such as the small sample size of the study and its retrospective design, which may lead to selection, recall, and information bias.

## Conclusions

The current study examined the effect of obesity and other factors on patients' length of stay post-appendectomy in Taif City, Saudi Arabia. While our study did not establish a significant correlation between body mass index BMI and extended hospital length of stay post-appendectomy, other variables, such as gender and appendectomy type, were significant factors associated with the hospital length of stay. Further studies with larger sample sizes are needed to validate these findings and explore other factors that may influence the outcomes of appendectomy in patients with appendicitis, such as the impact of obesity on long-term complications and recurrence rates.

## References

[1] Alharbi FM, Almutairi TS, Algayed HK, Alaskar MS, Alkhayari OA, Abaalkhail MK, Alshenghiti AM (2017). Predictors of length of stay, complications and patient’s satisfaction after appendectomy. Egyptian J Hosp Med.

[2] Peery AF, Crockett SD, Murphy CC (2019). Burden and cost of gastrointestinal, liver, and pancreatic diseases in the United States: update 2018. Gastroenterology.

[3] Bessoff KE, Choi J, Wolff CJ (2021). Evidence-based surgery for laparoscopic appendectomy: a stepwise systematic review. Surg Open Sci.

[4] Takami T, Yamaguchi T, Yoshitake H, Hatano K, Kataoka N, Tomita M, Makimoto S (2020). A clinical comparison of laparoscopic versus open appendectomy for the treatment of complicated appendicitis: historical cohort study. Eur J Trauma Emerg Surg.

[5] Towfigh S, Chen F, Katkhouda N, Kelso R, Sohn H, Berne TV, Mason RJ (2008). Obesity should not influence the management of appendicitis. Surg Endosc.

[6] Witt CE, Goldin AB, Vavilala MS, Rivara FP (2016). Effect of body mass index percentile on pediatric gastrointestinal surgery outcomes. J Pediatr Surg.

[7] Otake A, Horai M, Tanaka E (2019). Influences of total laparoscopic hysterectomy according to body mass index (underweight, normal weight, overweight, or obese). Gynecol Minim Invasive Ther.

[8] Lorio E, Ballard DH, Guarisco E, Hughes J, Griffen FD, Samra NS (2021). Appendectomy hospital stay: no difference in obese adult or pediatric patient length of stay compared to nonobese patients. Ochsner J.

[9] Prystowsky JB, Pugh CM, Nagle AP (2005). Appendicitis. Curr Probl Surg.

[10] Alvarado A (1986). A practical score for the early diagnosis of acute appendicitis. Ann Emerg Med.

[11] Delgado-Miguel C, Muñoz-Serrano AJ, Barrena Delfa S (2020). Influence of overweight and obesity on acute appendicitis in children. A cohort study. Cir Pediatr.

[12] Deugarte DA, Stark R, Kaji AH, Yaghoubian A, Tolan A, Lee SL (2012). Obesity does not impact outcomes for appendicitis. Am Surg.

[13] Garey CL, Laituri CA, Little DC, Ostlie DJ, St Peter SD (2011). Outcomes of perforated appendicitis in obese and nonobese children. J Pediatr Surg.

[14] Litz CN, Farach SM, Danielson PD, Chandler NM (2016). Obesity and single-incision laparoscopic appendectomy in children. J Surg Res.

[15] Knott EM, Gasior AC, Holcomb GW 3rd, Ostlie DJ, St Peter SD (2012). Impact of body habitus on single-site laparoscopic appendectomy for nonperforated appendicitis: subset analysis from a prospective, randomized trial. J Laparoendosc Adv Surg Tech.

[16] Mason RJ, Moazzez A, Moroney JR, Katkhouda N (2023). Laparoscopic vs open appendectomy in obese patients: outcomes using the American College of Surgeons National Surgical Quality Improvement Program database. J Am Coll Surgeons.

[17] Bhanderi S, Ain Q, Siddique I (2022). Demographic factors associated with length of stay in hospital and histological diagnosis in adults undergoing appendicectomy. Turk J Surg.

[18] Malik AA, Bari SU (2009). Conservative management of acute appendicitis. J Gastrointest Surg.

